# β-amyloid binds to microglia Dectin-1 to induce inflammatory response in the pathogenesis of Alzheimer's disease

**DOI:** 10.7150/ijbs.81900

**Published:** 2023-06-19

**Authors:** Xia Zhao, Jinfeng Sun, Li Xiong, Lingyu She, Liwei Li, Hao Tang, Yuqing Zeng, Fan Chen, Xue Han, Shiju Ye, Wei Wang, Xu Wang, Guang Liang

**Affiliations:** 1 Affiliated Yongkang First People's Hospital and School of Pharmacy, Hangzhou Medical College, Hangzhou, Zhejiang, 310012, China.; 2 Key Laboratory of Natural Medicines of the Changbai Mountain, Ministry of Education, College of Pharmacy, Yanbian University, Yanji, Jilin, 133002, China.; 3 Chemical Biology Research Center, School of Pharmaceutical Sciences, Wenzhou Medical University, Wenzhou, Zhejiang,325035, China.; 4 Oujiang Laboratory (Zhejiang Lab for Regenerative Medicine, Vision and Brain Health); Wenzhou Medical University, Wenzhou, Zhejiang,325035, China.

**Keywords:** β-amyloid, Dectin-1, Microglia, Neuroinflammation, Alzheimer's disease

## Abstract

Microglia-mediated neuroinflammation is closely related to the development of Alzheimer's disease (AD). In the early stages of the inflammation response, pattern recognition receptors (PRRs) play a key role in clearing damaged cells and defending against infection by recognizing endogenous and exogenous ligands. However, the regulation of pathogenic microglial activation and its role in AD pathology remains poorly understood. Here we showed that a pattern recognition receptor called Dectin-1, expressed on microglia, mediates the pro-inflammatory responses of beta-amyloid (Aβ). Knockout of Dectin-1 reduced Aβ1-42 (Aβ_42_)-induced microglial activation, inflammatory responses, and synaptic and cognitive deficits in Aβ_42_-infused AD mice. Similar results were obtained in the BV2 cell model. Mechanistically, we showed that Aβ_42_ could directly bind to Dectin-1, causing Dectin-1 homodimerization and activating downstream spleen tyrosine kinase (Syk)/nuclear factor-κB (NF-κB) signaling pathway to induce the expression of inflammatory factors and, in turn, AD pathology. These results suggest the important role of microglia Dectin-1 as a new direct receptor for Aβ_42_ in microglial activation and AD pathology and provide a potential therapeutic strategy for neuroinflammation in AD.

## Introduction

Alzheimer's disease (AD) is characterized by a progressive loss of memory and cognitive impairment. Although multiple pathological theories have been proposed, the underlying mechanism remains unclear, leading to ineffective treatments. Neuroinflammation is an early pathological change that drives the progression of AD 1. Microglia, the resident mononuclear phagocytes of the central nervous system (CNS), are the main executors of immune functions in the brain 2. Genome-wide association studies have shown that many AD risk genes are highly expressed in microglia and play important roles in the occurrence and development of AD 3. Therefore, microglia are crucial in the early stages of AD and may be potential therapeutic targets. Beta-amyloid (Aβ) deposition is one of the most important pathological features of AD and is produced by the cleavage of amyloid precursor protein (APP) by β-secretase and γ-secretase 4. Among amyloid-forming fragments, Aβ_42_ is the most toxic and easily aggregates to form amyloid plaques 5. Aβ can activate microglia, resulting in production of inflammatory factors such as tumor necrosis factor (TNF)-α, interleukin (IL)-6, and IL-1β 6. Injecting Aβ_42_ into the mouse hippocampus can induce microglial activation and extensive inflammatory responses, causing significant effects on the central nervous system 7, 8. In the brains of patients with AD, approximately 80% of the "amyloid plaque" structures are covered by activated microglia 9. These studies indicate a key role for Aβ in microglial activation, neuroinflammation, and AD development. However, the precise mechanism and direct receptors by which Aβ induces inflammation in microglia are not fully understood.

Pattern recognition receptors (PRRs) play an important role in clearing damaged cells and defending against infection by recognizing endogenous and exogenous ligands 10. Dectin-1 (encoded by *Clec7a* gene), a member of the C-type lectin family of PRRs, is expressed selectively in myeloid-monocytic lineage cells, including microglia 11. Dectin-1 contains an extracellular C-like lectin domain (C-lectin domain), a transmembrane domain, and an intracytoplasmic tyrosine activation motif (ITAM). When Dectin-1 is activated by ligands, intracellular ITAM recruits spleen tyrosine kinase (Syk) to induce Syk phosphorylation, which further activates NF-κB to induce pro-inflammatory gene transcription 12. The C-lectin domain in the extracellular segment plays the role of recognizing and binding ligands 13. Originally, Dectin-1 was found to recognize β-glucans in fungal pathogens and elicit antifungal pro-inflammatory immune responses. Recent studies have shown that in addition to classical β-glucan ligands, Dectin-1 is also involved in non-pathogen-mediated sterile inflammation 14. In the central nervous system, Dectin-1 is significantly increased upon exposure to various stimuli such as ischemia and injury 15. In models of intracerebral hemorrhage, Dectin-1 has been shown to cause macrophage polarization, and the activated Syk/NF-κB pathway contributes to brain injury 15. Dectin-1 antagonist treatment significantly reduces the number of activated microglia and the level of inflammation in ischemic brain tissue and OGD/R-treated microglia 16, 17. In addition, activation of Dectin-1 leads to macrophage-mediated axonal damage, whereas blockade of Dectin-1 reduces inflammatory macrophage-mediated axonal damage after spinal cord injury 18. These studies suggest that Dectin-1 plays an important role in neurological diseases. However, whether Dectin-1 is involved in Aβ-induced neuroinflammation and AD pathology is largely unknown. More importantly, endogenous ligands that activate Dectin-1 in the brain under sterile inflammatory conditions have not been identified.

In this study, we investigated the expression and function of Dectin-1 in an Aβ_42_-infused model of AD. Our results showed that the knockout of Dectin-1 improved neuroinflammation, cognitive function, and neurological damage in Aβ_42_-infused mice. Importantly, our detailed mechanistic studies in microglia revealed that Aβ directly binds to Dectin-1 to initiate Syk/NF-κB signaling and induce inflammatory cytokines. This study identified Dectin-1 as a novel non-classical Aβ receptor and suggested that it plays an important role in neuroinflammation and AD pathology.

## Materials and Methods

### General Reagents

Amyloid beta-peptide (1-42) (PA4391) was purchased from Ontores Biotechnologies (Zhejiang, China). Recombinant human Dectin-1 (rhDectin-1) was purchased from Sino Biological (10215-HNCH; Beijing, China). HA-tag (51064-2-AP) and Flag-tag (20543-1-AP) were ordered from Proteintech (Shanghai, China). Biotin (D8150) and Biotinylated-Aβ_42_ (Bio-Aβ_42_) was obtained from GL Biochem (Shanghai, China). Penicillin/Streptomycin and Opti-MEM were ordered from Gibco (Carlsbad, CA, USA). Annexin V-FITC/PI Apoptosis Detection Kit was purchased from BD Biosciences (San Diego, CA, USA). Bovine serum albumin (BSA), Dulbecco's modified Eagle's medium (DMEM) and Dimethyl sulfoxide (DMSO) were ordered from Sigma (St. Louis, MO, USA). MTT powder, reactive oxygen species assay kit (ROS), JC-1 assay kit, RIPA lysis buffer and Hoechst 33258 were bought from Beyotime Institute of Biotechnology (Shanghai, China). PVDF membrane, ECL Plus and Western Blot Marker were bought from Bio-Rad. The list of antibodies used and their source is presented in the supplementary information Table S1. Sequence Information of β-Amyloid (1-42) used in this study was shown in the Supplementary Information Table S2.

### Experimental AD models and treatment

The experimental protocol was approved by Hangzhou Medical College Animal Ethics Committee (2022-001). D1KO male mice (18-22g) on a C57BL/6 background and wild type male C57BL/6 mice (18-22g) were ordered from GemPharmatech (Nanjing, China). The dectin-1 knockout allele lacks sequences corresponding to the cytoplasmic tail region, transmembrane region and stalk region of the Dectin-1 locus (*Clec7a*). All the mice were housed in Hangzhou Medical College Animal Research Center under 22-24°C, 60-65% humidity, 12 hours/dark-light cycle, and fed a standard rodent diet. Before initiating the studies, all the mice were acclimatized in the laboratory for at least 2 weeks. We used two AD models in this study: Aβ_42_ infusion AD Model and APP/PS1 (APPswe, PSEN1dE9) AD model.

#### Aβ_42_ infusion AD Model

Eight-week-old Wide Type C57BL/6 mice (WT mice) and Dectin-1^-/-^ on Wide Type background mice were randomly divided into four groups: WT (WT mice controls, n = 10), Aβ_42_ (Aβ_42_-infused WT mice; n = 10), D1KO (Dectin-1^-/-^ mice controls; n = 10), and D1KO+Aβ_42_ (Aβ_42_-infused Dectin-1^-/-^ mice; n = 10). Aβ_42_ peptide was incubated at 37°C to aggregate for one week before use 19. All the mice were anesthetized with an intraperitoneal injection of 1% pentobarbital (40 mg/kg) and fixed to a stereotactic apparatus. A single injection of the aggregated form of Aβ_42_ (5μg/μL) was administered unilaterally into the right hippocampal CA1 molecular layer of the mice (2μL per mouse). The following injection site coordinates were determined based on the Paxinos and Franklin atlas 20. The bregma is 0; the rear opening is 2.0 mm, the side opening is ± 1.5 mm, and the depth is 1.5 mm. After injection, the needle was kept in place for another 2 minutes and then pulled out slowly.

#### APP/PS1 (APPswe, PSEN1dE9) AD model

APP/PS1 double transgenic mice were obtained from The Jackson Laboratory [B6; C3-Tg (APPswe, PSEN1dE9)85Dbo/Mmjax]. These mice are expressing a mutant human presenilin 1 (PS1-dE9) and a chimeric mouse/human amyloid precursor protein (Mo/HuAPP695swe), which directly target neurons in the central nervous system. Both mutations have been linked to early-onset Alzheimer's disease. These mice are widely used in studies of Alzheimer's disease 21. Six-month-old Wide Type mice (n = 3); Six-month-old APP/PS1 mice (n = 3) were used for Western blotting and IF to detect Dectin-1 expression.

### Morris water maze (MWM)

To test the effect of Dectin-1 on the memory and learning abilities of AD model mice, the Morris water maze was performed. Briefly, mice were tested for place navigation on 4 consecutive days followed by a spatial probe test (without platform) on the 5^th^ day 22. During the place navigation tests the platform was placed 1 cm below the water surface in the middle of the target quadrants. The time and movement route of the mice to find the platform were recorded. The above operation was repeated daily from the 2^nd^ until the 4^th^ day. On the 5^th^ day, the platform was removed and the mice were allowed to swim freely for 60 s for spatial probe experiments. The time spent in the target quadrant and the number of crossings platform were recorded 23. All data collection and processing were done by image automatic monitoring and processing system (VisuTrack, Shanghai China).

### New Object Recognition (NOR)

The bottom surface of the mouse experimental device is a square of 25 × 25 cm, and the four walls are 40 cm high. Before the experiment, the mice of each group were moved to the test room to acclimate to the test environment. On the first day, two identical objects A were placed in the experimental setup. After the mice were put in, the video equipment was turned on to record the contact between the mice and the two objects, including the number of times they touched the objects and the time spent exploring within 2-3 cm from the objects. The test duration was 5 minutes. After 24 hours, replace one of the A objects with B objects, and record with video equipment for 5 minutes. Then analyze the cognition of the mice: if the mice have poor cognitive ability, there is no difference in the exploration of new and old objects; if the mice have normal cognitive ability, the exploration of new objects is longer than that of old objects. The cognitive index (recognition index, RI) is calculated as: RI = new object / (new object + old object) × 100 %.

### Immunofluorescence (IF)

After post-fixation in 4% PFA for approximately 24 hours, the paraformaldehyde was replaced by 20% sucrose. After the samples sunk to the bottom of the container, the 20% sucrose was replaced by a 30% sucrose solution until the samples sinking. The brains were cut at a thickness of 20 μm using a low temperature thermostat (Leica CM3050, Germany). Each section was dried for 20 minutes and then washed with 1xPBS for three times. Non-specific binding was blocked with 10% bovine serum albumin for 60min at room temperature. After that, tissue sections were incubated with the corresponding primary antibodies at 4°C overnight. Next day, all the sections were incubated with appropriate secondary antibody for 1h at room temperature. All incubations were followed by three washes in PBS for 15 min. Nuclei were stained with DAPI (Sigma, D6578), and the images were acquired with a Nikon A1 confocal microscope.

### ELISA assay

After behavior test, blood samples from each group of mice were collected. Serum was obtained by centrifuge at 12000 rpm for 10 min and the levels of TNF-α and IL-1β were measured using ELISA kits. Briefly, samples were added to the wells with a biotin-conjugated antibody specific to TNF-α or IL-1β. Then, Avidin conjugated to Horseradish Peroxidase (HRP) was added to each microplate well and incubated. Then, TMB substrate solution was added and incubated, those wells that contain TNF-α or IL-1β, biotin-conjugated antibody and enzyme-conjugated Avidin exhibited a change in color. Finally, stop solution was added to stop the reaction and the color change were measured at a wavelength of 450 nm by a microplate reader. The concentration of TNF-α or IL-1β in the samples were then determined by comparing the OD value of the samples to the standard curve.

### Immunocytochemistry (ICC)

Properly treated BV2 cells were fixed in 4% paraformaldehyde (PFA) for 15-20 min. The BV2 cells were then incubated with 1x PBS containing 0.1% Triton X-100 (PBST) for 20 min to increase membrane permeability. After block with 1% BSA for 1 h at room temperature, the primary antibody (1:100) was added and incubate overnight at 4 °C. Next day, the cells were washed three times with 1xPBS and incubated with fluorophore conjugated secondary antibody (1:500) for 2h. Finally, one drop of antifade mounting medium with DAPI (P0131, Beyotime) was added to stain the Nuclei. Then, sections were observed and analyzed under a Nikon A1 confocal microscope.

### TUNEL staining

To test cellular apoptosis, TUNEL assay was performed (C1098, Beyotime, Shanghai, China). For cells: Properly treated BV2 cells were fixed with 4% PFA for 20 min and washed with PBST for 3 times. Then cells were incubated with TUNEL test solution (5 μl of TdT enzyme and 45 μl of fluorescent labeling solution) under dark condition for 1h. After washing with 1xPBS for three times, nuclei were stained with DAPI. The apoptotic cells (green fluorescence) were observed under fluorescent microscopy and calculated as a percentage of the total number of cells. For brain tissue: Processed tissue samples were incubated with 50 μl of TUNEL reaction mixture for 1h at 37℃ under dark conditions. Color developed in DAB coloring solution. Apoptosis index (brown color) in the hippocampus area were measured using this formula: apoptosis index (%) = (apoptotic neurons / total neurons) × 100%.

### Microglia Phagocytosis and Clearance Assay

BV2 cells were seeded in 12-well plates and divided into four groups: Ctrl, Aβ_42_, siD1 and Aβ_42_+siD1 group. Cells were transfected with Dectin-1 interference plasmid or control plasmid with liposome 2000 for 24 h. Then incubated with/without 20 μM FITC-Aβ_42_ for 2 h. After incubation, the medium containing FITC-Aβ was removed, and the cells were washed three times with 1xPBS. BV2 cells were collected by centrifugation at 3000rpm for 5min, and resuspended in ice-cold 1xPBS solution. The phagocytosed Aβ_42_ content in BV2 cells was analyzed by flow cytometry, and PBS solution (PH4.4) was added to the sample for 1 min incubation before flow cytometry analysis to quench the cell surface-bound Aβ_42_. To observe the effect of Dectin-1 on the degradation of Aβ_42_ by BV2 cells, extracellular FITC-Aβ was removed after the cells were incubated with FITC-Aβ for 2 h. Cells were cultured in the FITC-Aβ-free medium for another 24 hours. Collect the cells and detect the level of intracellular Aβ by flow cytometry.

### Molecular docking

The crystal structure of murine dectin-1 and human amyloid beta-peptide (1-42) were downloaded from RCSB Protein Data Bank (http://www.rcsb.org/) and the PDB ID for murine dectin-1 was 2BPD^1^ while 6SZF^2^ was for human amyloid beta-peptide (1-42). Protein-protein docking in ClusPro^3^ was used for molecular docking simulation of dectin-1 and amyloid beta-peptide. For protein docking, 6SZF is set as ligand and 2BPD as receptor. The ligand was rotated with 70,000 rotations, among which 1000 rotation/translation combinations that have the lowest score was chosen. Then, a greedy clustering of these 1000 ligand positions with a 9 Å C-alpha RMSD radius was performed to find the ligand positions with the most “neighbors” in 9 Å, i.e., cluster centers. The top ten cluster centers with most cluster members were then retrieved and inspected visually one by one. The most likely models of the complex are selected on the basis of cluster population. The intermolecular contacts from the most probable poses were further evaluated. The models of the complex and interface residues were analyzed using MOE^4^. Molecular graphics were generated by PyMOL. The summary of interactions between dectin-1 and amyloid beta-peptide is listed in Table S3.

### Surface Plasmon Resonance (SPR) analysis

The potential binding of Aβ_42_ to recombinant human Dectin-1 (rhDectin-1, Sino Biological lnc, 10215-HNCH) was measured using Biacore T200 Protein Interaction Assay system (GE Healthcare) with a CM7 sensor chip (29-1470-20). Fifty μg rhDectin-1 protein was dissolved in 50 μL PBS Buffer (pH 7.4), and then amine coupling kit (Fortebio) was used to immobilize rhDectin-1 on the chip. Different doses of Aβ_42_, including 62.5, 31.25, 15.625, 7.8, 3.4, 1.7, 0.85 μM were prepared with running buffer (PBS containing 0.02%Tween20). The sample plates and sensor were placed in the instrument. During the association phase, the interactions were determined at a flow rate of 30 μL/min for 180 s, then the dissociation phase at 25°C for 250s. Data were analyzed using Biacore T200 manager software. The binding kinetic parameters were calculated by global fitting of the kinetic data from various concentrations of Aβ_42_ using a 1:1 Langmuir binding model.

### RNA-seq and bioinformatics analysis

*RNA* sequencing was performed by Wuhan *Huada Gene Technology Co., Ltd*. Two independently prepared *RNA* samples from each group *were* prepared for *RNA*-*Seq*. The sequencing data was filtered with SOAPnuke (v1.5.2). The clean reads were mapped to the reference genome using HISAT2 (v2.0.4). Then, Ericscript (v0.5.5) and rMATS (V3.2.5) were used to analyze the fused genes and differential splicing genes (DSGs), respectively. Bowtie2 (v2.2.5) was used to align the clean reads to the gene set, a database for this organism built by BGI (Beijing Genomic Institute in Shenzhen), which included known and novel coding transcripts. Gene expression levels were calculated using RSEM (V1.2.12). The heatmap was drawn by pheatmap (v1.0.8) according to the gene expression in different samples. The DESeq2 (v1.4.5) with Q value ≤ 0.05 was used for differential expression analysis. The sources of software were summarized in Table S4.

### GEO Database Analysis

The Gene Expression Omnibus (GEO, http://www.ncbi.nlm.nih.gov/geo) contains the original data of many diseases for users to download and research. In the present study, gene expression profile datasets GSE173955 were obtained by searching GEO database in normal people and AD patients. The* Clec7a* mRNA expressions between brain tissue of AD patients (N=8) and non-AD patients (N=10) were analyzed.

### Pull-down Assay

100µL of 20 μM biotinylated- Aβ_42_ was added to streptavidin-agarose beads (50 μL) and incubated for 45 min at 4°C with biotin alone as a control. Lysates prepared from microglia cells and mouse brain tissues were then added to the streptavidin-agarose beads with biotinylated- Aβ_42_. The mixture was incubated for 24h at 4°C under gentle rocking. Samples were then spun and washed 3 times. Then, 5x loading buffer was added in the elution buffer and boiled for 10min, and the samples were used for western blot analysis. Total lysates were used as an input control.

### Real-time qPCR

Total RNA was isolated from cultured BV2 cells and brain tissues using TRIzol reagent (Thermo Fisher, 15596026) by standard techniques. Total RNA concentration was measured by a spectrophotometer. Then, PrimeScript RT-reagent Kit (Takara, RR037A) was used for cDNA synthesis. Real-time PCR amplification reactions were carried out on CFX96 Touch Real-Time PCR Detection System (Bio-Rad) using TB Green Premix Ex Taq II (Takara; RR820A). Quantification of the mRNA expression was calculated by 2^ΔΔCt^ method with *GAPDH* normalization. Primer sequences used in this study were presented in Supplementary Table S2.

### Western blot analysis

The cultured BV2 cells or brain tissue samples under different treatments were lysed in ice-cold RIPA lysis buffer (P0013B; Beyotime Biological Technology, Shanghai, China) supplemented with a protease phosphatase inhibitor cocktail and the protein concentration was tested using a BCA protein assay kit. Samples with the same concentration of proteins were loaded on 12% SDS-PAGE gels, then transferred to 0.22 μm PVDF membrane at 300 mA for 2h. The PVDF membranes containing protein bands were blocked with 3% BSA for 1 h at room temperature and incubated with selective primary antibodies (1:1000) overnight at 4 ℃. Following day, the primary antibody was washed with 1×TBST (Tris-buffered saline containing 0.05% Tween20) thrice, and incubated with horseradish peroxidase (HRP)-conjugated secondary antibody for another 2h at room temperature. After exposure with BCL, the intensity of bands were quantified using Image J software.

### Statistical analysis

Data analysis was performed using GraphPad Prism 8.0 statistical software (GraphPad software, Inc., San Diego, CA, USA). Each experiment was carried out in triplicates and all the data was expressed as mean ± SEM. The statistical differences between multiple groups was determined using one or two-way ANOVA followed by Tukey's post-hoc test (α = 0.05) to assess the difference between any two groups. For the MWM test, escape latency times in the hidden platform trial were analyzed via two-way ANOVA of repeated measures.

## Results

### Dectin-1 is up-regulated in AD model mice

**Figure 1:**
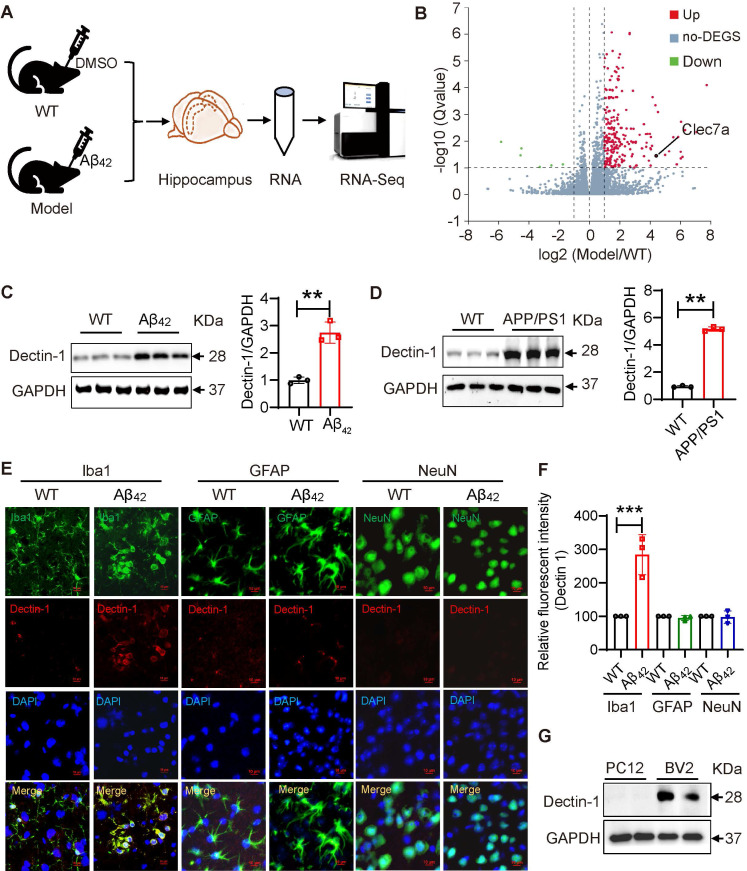
** Dectin-1 is up-regulated in AD model mice. (A)** RNA-seq analyses using hippocampus from the brains of C57 following DMSO infusion or C57 following Aβ_42_ infusion for two weeks. N=6, 3 mice per group. **(B)** Bioinformatic analysis of RNA-seq. **(C)** Protein levels of Dectin-1 in the hippocampal tissue following Aβ_42_ infusion. **(D)** Protein levels of Dectin-1 in the hippocampal tissues of APP/PS1 mice. **(E)** Representative dual-immunofluorescence staining of Microglia marker Iba1 (green), Astrocyte marker GFAP (green), Neuron Marker (NeuN) and Dectin-1 (red) in the hippocampal tissue of Aβ_42_ infused mice. Sections were counterstained with DAPI (blue) [scale bar = 10 μm]. **(F)** Quantification analysis of Dectin-1 in E. **(G)** Western blot level of Dectin-1 in mouse bone marrow-derived macrophages (PC12), microglia cell line (BV2).

In recent years, substantial evidence has shown that Dectin-1 is involved in neurological pathology 24. Through GEO database analysis, we observed that the Dectin-1 mRNA level (CLEC7a) was significantly elevated in patients with AD compared to patients without AD (Figure S1). We further analyzed gene expression changes in the hippocampus of mice with or without Aβ_42_ injection using RNA Sequencing (RNA-seq) (Figure 1A). In this study, we used aggregated Aβ_42_, as aggregated Aβ is more toxic. First, Aβ_42_ was prepared as a 10 mM stock solution in sterile DMSO, then incubated at 37°C for 7 days before use. The aggregation state of Aβ_42_ in this experiment was detected by non-denaturing gel and Coomassie brilliant blue staining. Obtained result showed that Aβ_42_ after 37°C aggregation is a low molecular weight (LMW) oligomer (Figure S2). Immunofluorescence staining of APP/β-amyloid and the microglia marker Iba1 in the mouse hippocampus confirmed that Aβ_42_ infusion successfully induced AD pathology (Figure S3A). Similar results were obtained in the western blot assay (Figure S3B). The data also showed that Aβ_42_ injection increased the levels of the inflammatory factors tumor necrosis factor-α (TNFα) and IL-1β in the hippocampal tissue (Figure S3C). Interestingly, RNA-seq analysis showed that Dectin-1 mRNA levels were also significantly increased in the hippocampal tissue of Aβ_42_-infused mice compared to those in the WT group (Figure 1B). Western blot assay of mouse hippocampal tissues validated the increased Dectin-1 level following Aβ_42_ challenge (Figure 1C). To further confirm this, we used 8-month-old APP/PS1 mice as another AD model. As shown in Figure 1D, Dectin-1 protein levels were also significantly increased in the hippocampal tissues of APP/PS1 mice compared to WT mice (Figure 1D).

Dectin-1 has been reported to be highly expressed by myeloid-lineage cells such as macrophages 25. Therefore, we expected microglia to be the primary source of increased Dectin-1 in brain tissue. To confirm this, we double-labeled microglia markers (Iba1), astrocyte markers (GFAP), Neuron markers (NeuN) and Dectin-1 in Aβ_42_ injection models, respectively. As shown in Figure 1E, there is almost no expression of Dectin-1 in neurons, a small amount of Dectin-1 expression in astrocytes, and the highest relative expression in microglia, suggesting that microglia are the main cell type expressing Dectin-1 in the hippocampus (Figure 1E-F). We then examined Dectin-1 expression in the PC12 and BV2 cell lines. As expected, Dectin-1 was expressed in BV2 cells, but not in PC12 cells (Figure 1G). Taken together, these results further validate that Dectin-1 is expressed in microglia and is significantly increased in Aβ_42_ infusion and APP/PS1 mouse models, suggesting that Dectin-1 in microglia may play an important role in the pathology of AD.

### Dectin-1 knockout alleviates cognitive impairment in Aβ_42_ infusion mice

**Figure 2:**
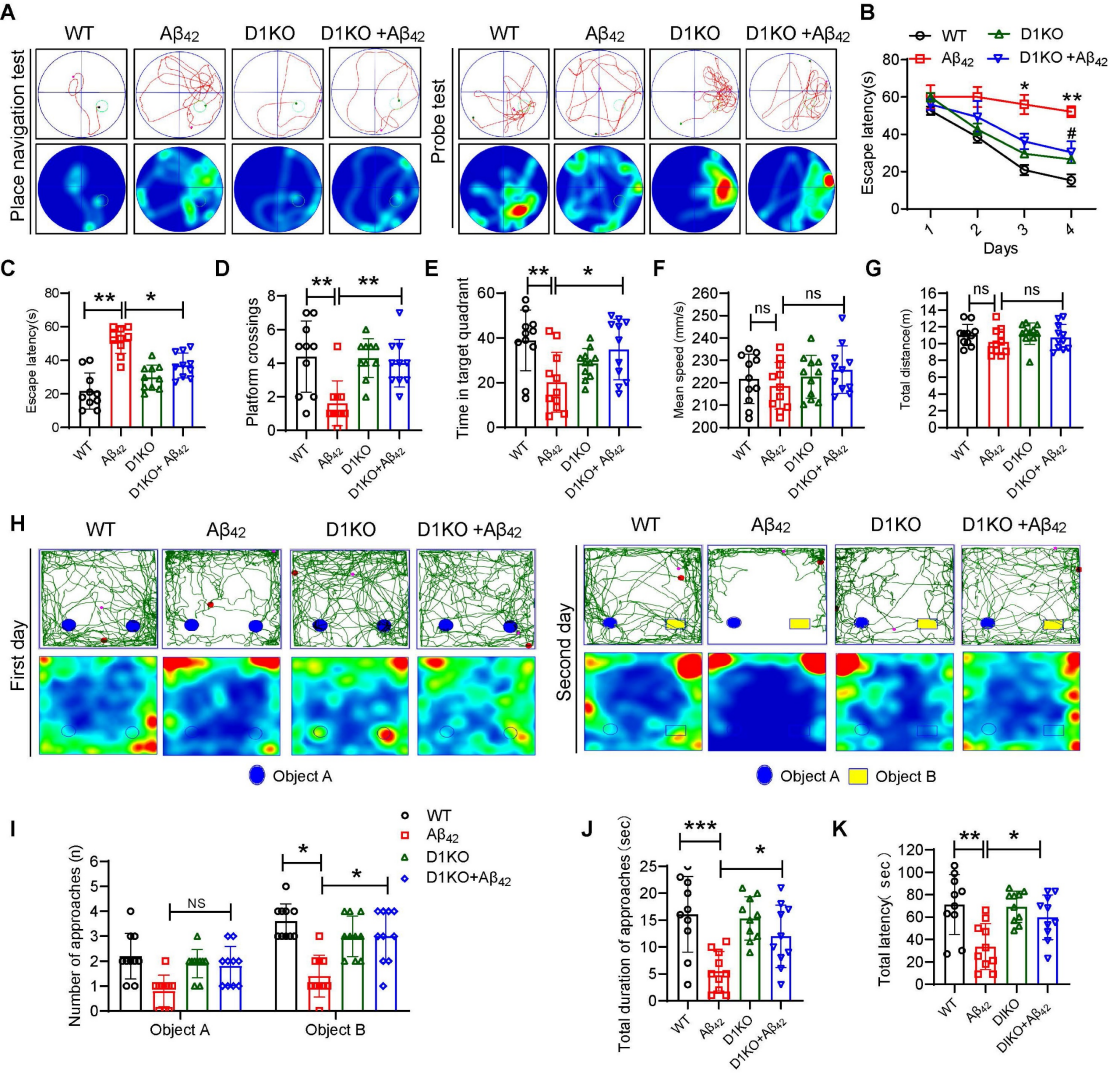
** Dectin-1 knockout restore cognitive functions of Aβ_42_ infusion model mice. (A)** Representative average escape latency curves during place navigation test (four trials per day, during four consecutive days) and probe tests on day 5 without platform. N=40, N=10 mice per group.** (B)** Time course of escape latency, defined as the time taken to find the hidden platform. **(C)** Escape latency of all four groups on day 4. **(D)** The number of platform crossings. **(E)** The time in target quadrant. **(F)** Average swimming speed of mice in each group. **(G)** Total swimming distance of mice in each group. **(H)** Representative curves during novel object recognition test in each group. **(I)** Number of approaches in each group. **(J)** Total during of approaches in each group. **(K)** Total latency of all four groups.

To assess the role of Dectin-1 in the development of AD, Dectin-1 knockout (D1KO) mice were used. We first verified that Dectin-1 was knocked out in the brain tissue of D1KO mice (Figure S4A-B). Both WT and DIKO mice were injected with or without Aβ_42_ to develop the AD model or control groups, respectively (Figure S5A). The Morris water maze (MWM) and novel object recognition assay (NOR) were used to assess learning and memory abilities in four groups: WT (wild-type), Aβ_42_ (Aβ_42_ injection), D1KO (Dectin-1**^-/-^**), and D1KO+Aβ_42_. The movement trajectory of each group of mice was recorded using the Morris water maze test (Figure 2A). The average escape latency of mice injected with Aβ_42_ was significantly increased. However, the average escape latency of D1KO mice was significantly lower than that of mice in the Aβ_42_ infusion group (Figure 2B-2C). After removing the platform, mice in the D1KO group spent more time crossing the platform and stayed in the target quadrant compared to mice in the Aβ_42_ group (Figure 2D-2E). In addition, there were no significant differences in swimming speed and total distance between the different groups of mice (Figure 2F-2G), excluding the potential influence of differences in exercise capacity on this experiment. These results suggest that Dectin-1 knockout significantly alleviates cognitive impairment induced by Aβ_42_ infusion. We further verified our results using a novel object recognition assay. The representative curves are shown in Figure 2H. We recorded the number of novel object explorations (Figure 2I), total time spent exploring novel objects (Figure 2J), and total latency to touch novel objects (Figure 2K) of the mice in each group. The results showed that the exploration of novel objects increased in the D1KO group compared to that in the Aβ_42_ infusion group. These data indicate that knockout of Dectin-1 significantly improves cognitive impairment in Aβ_42_ infusion model mice.

### Dectin-1 knockout improves neuropathology in Aβ_42_ infusion mice

**Figure 3:**
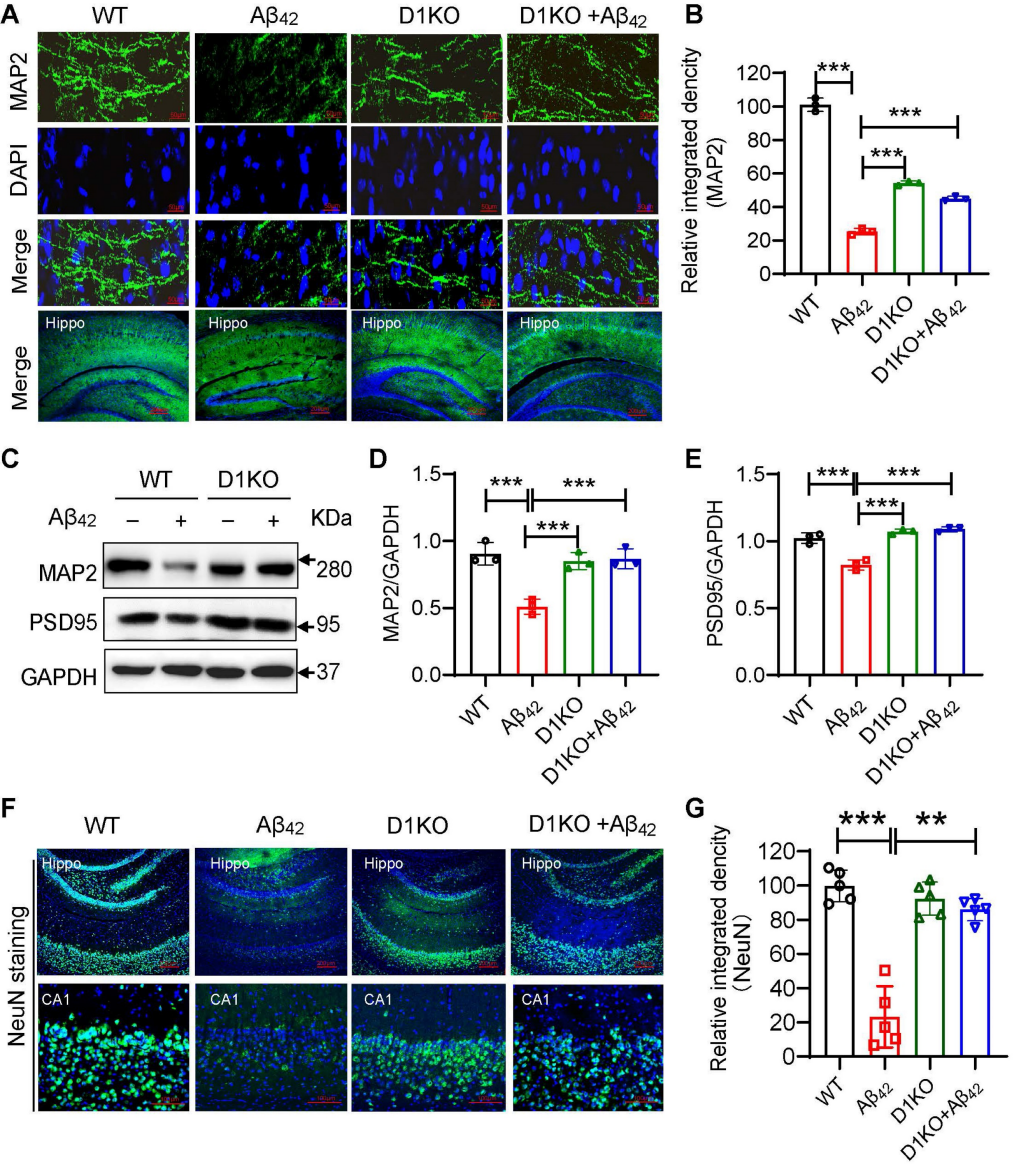
** Dectin-1 knockout improves neuropathology of Aβ_42_ infusion mice. (A)** Representative immunofluorescence staining of MAP2 (green) in brain tissues of WT and Aβ_42_ infusion mice. Sections were counterstained with DAPI (blue) [scale bar = 50 μm]. **(B)** Quantification of MAP2 staining. **(C)**Representative western blot analysis of MAP2 and PSD95 in hippocampal tissue lysates from WT and Aβ_42_ infusion mice. **(D)** Quantification and statistical analysis of MAP2 in C.** (E)** Quantification and statistical analysis of PSD95 in C. **(F)** IF of NeuN staining: Hippo [scale bar = 200 μm], CA1[scale bar = 100 μm] in brain tissues of WT and Aβ_42_ infusion mice. **(G)** Quantification of NeuN staining.

Damage to brain synaptic structure and functional plasticity is a major pathology of AD, resulting in a decline in the learning and memory abilities of patients with AD 26. Therefore, we examined synaptic plasticity. IF staining of the mouse hippocampus showed a significant loss of synapses in the Aβ_42_ infusion mice, while the number of neurite cytoskeleton microtubule-associated protein 2 (MAP2)-expressing cells in the D1KO+Aβ_42_ group was significantly increased (Figure 3A-3B). Western blotting validated the change in the expression of MAP2 and postsynaptic density protein 95 (PSD95) (Figure 3C-3E). Synaptic plasticity is important for maintaining neuronal function and activity in the CNS 27. We tested the number of neuronal cells in the hippocampus using NeuN staining. As shown in Figure 3F-3G, Dectin-1 deficiency reduced the number of apoptotic neurons in the Aβ_42_-infused mouse hippocampus.

### Dectin-1 knockout alleviates Aβ_42_-induced microglia activation and inflammatory response

**Figure 4:**
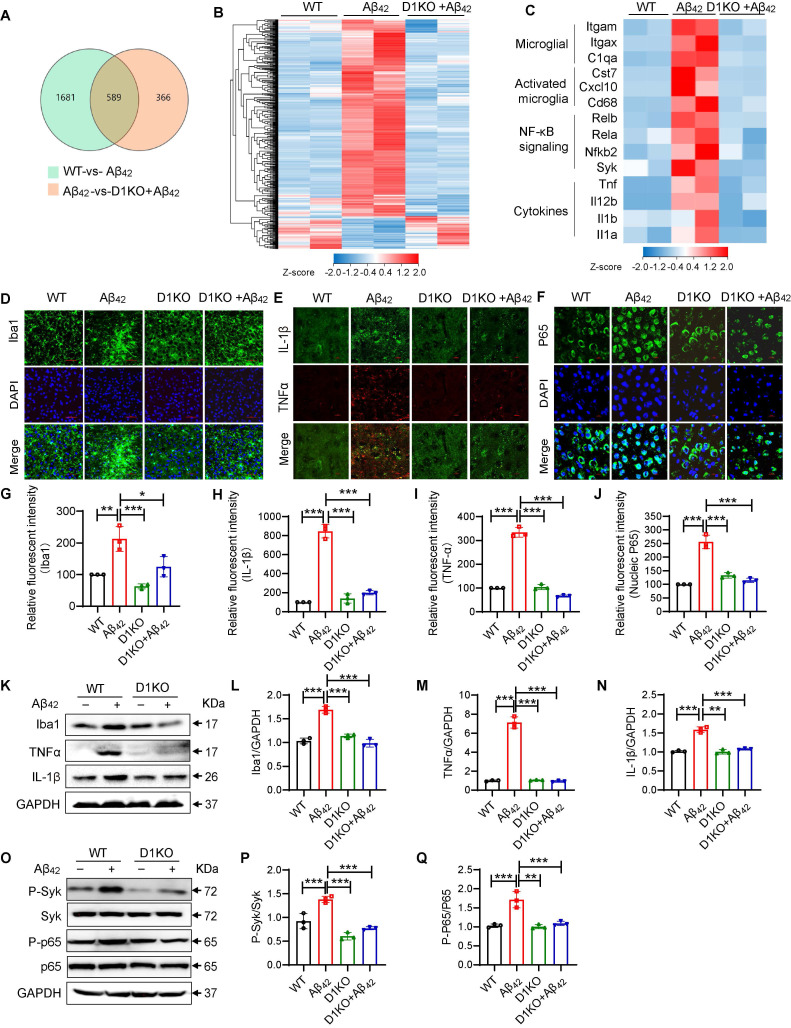
** Dectin-1 mediates microglia activation and inflammatory response in brain tissue. (A)** Venn diagram depicting expressed genes in WT-vs- Aβ_42_ and D1KO-vs- Aβ_42_. **(B)** Heatmap shows four groups of expressed genes that expressed differentially between 8-week-old WT, Aβ_42_, D1KO, and D1KO+ Aβ_42_ mice (P < 0.05). **(C)** Heatmap of microglia markers, markers in activated microglia, NF-κB signaling, and cytokines from these four groups of mice.** (D)** Representative immunofluorescence staining of Iba1(green) in brain tissues of WT and Model (Aβ_42_ infusion) mice. Sections were counterstained with DAPI (blue) [scale bar = 50 μm]. **(E)** Representative double-immunofluorescence staining of TNFα (red) and IL-1β (green). Slides were counterstained with DAPI (blue) [scale bar = 10 μm].** (F)** Representative immunofluorescence staining of NFκB (green) in brain tissues of WT and Aβ_42_ infusion mice. Sections were counterstained with DAPI (blue) [scale bar = 10 μm]. **(G)** Quantification of Iba1 staining in D. **(H-I)** Quantification of IL-1β and TNFα staining in E. **(J)** Quantification of NFκB staining in F. **(K-N)** Representative western blot analysis of Iba1, TNFα, and IL-1β in hippocampus tissue lysates.** (O-Q)** Representative western blot analysis of p-Syk, Syk, P-NFκB65, and NFκB65 in mouse brain tissue lysates.

To further investigate the molecular mechanisms underlying the functions of Dectin-1 in AD, we isolated mRNA from all three groups of mice (WT, Aβ_42_ and D1KO+ Aβ_42_) and performed RNA-seq analysis. A total of 589 genes were identified (Figure 4A). Notably, microglial D1KO restored the levels of most of the upregulated genes and increased the levels of many of the downregulated genes (Figure 4B). To further characterize the inflammatory changes, we analyzed the levels of microglial markers, innate immune signaling genes, and inflammatory cytokines. As shown in Figure 4C, Dectin-1 knockout significantly reduced the increase in microglial markers (Itgam, Itgax, and C1qa), activated microglia markers (Cst7, Cxcl10, and CD68), NF-κB signaling genes (Rel1, Rel2, Nfkb1, Nfkb2, and Syk), and cytokines (Il1b, Tnfa, Il1a, and Il12b), suggesting inhibition of microglial activation and inflammatory activation. Similarly, Dectin-1 knockout significantly reduced the number of Iba1-positive cells (Figure 4D and 4G). We also tested the effect of Dectin-1 on the inflammatory response and NF-κB p65 using immunofluorescence staining (Figure 4E-4F and 4H-4J), western blotting (Figure 4K-4N) and ELISA (Fig S5A-5B). The data showed that injection of Aβ_42_ resulted in increased release of the pro-inflammatory cytokines TNFα and IL-1β in mouse hippocampal tissues, while Dectin-1 deficiency blocked these changes. We next explored whether the canonical downstream Syk-NF-κB pathway of Dectin-1 12 was activated by Aβ_42_ injection. Hippocampal tissues from WT mice injected with Aβ_42_ showed increased p-Syk, phosphorylated NF-κB p65, and nuclear NF-κB p65 translocation, indicating activation of Syk/NF-κB by Aβ_42_ infusion (Figure 4O-4Q). Dectin-1 knockout mice reduced Aβ_42_-induced Syk/NF-κB activation.

The anti-inflammatory actions of Dectin-1 deletion may result from two possible mechanisms: 1) Dectin-1 mediates the pro-inflammatory signaling pathway of Aβ and 2) Dectin-1 deletion reduces Aβ levels by promoting Aβ clearance in the hippocampus. Therefore, we measured the effect of Dectin-1 on Aβ clearance in the hippocampus. As shown in Figure S6B-6D, Dectin-1 deficiency did not affect the level of Aβ plaques in the mouse hippocampus. To verify the effect of Dectin-1 on phagocytosis and clearance of Aβ_42_, we linked Aβ_42_ with a FITC label (FITC-Aβ_42_). We treated Dectin-1-deficient BV2 cells with FITC-Aβ for an appropriate time, and then used flow cytometry to detect the phagocytosis and clearance of Aβ_42_ by Dectin-1. The obtained results found that the Dectin-1 deficiency did not affect the fluorescence intensity of Aβ (Fig S6E-6F), indicating that Dectin-1 does not mediate Aβ phagocytosis and clearance in microglia. Taken together, Aβ_42_ activates Dectin-1 to induce Syk phosphorylation and NF-κB activation, resulting in an increase in the levels of pro-inflammatory factors, rather than Aβ phagocytosis and clearance in microglia.

### Aβ_42_ activates the Dectin-1-Syk signaling pathway by binding to Dectin-1 and inducing its homodimerization

**Figure 5:**
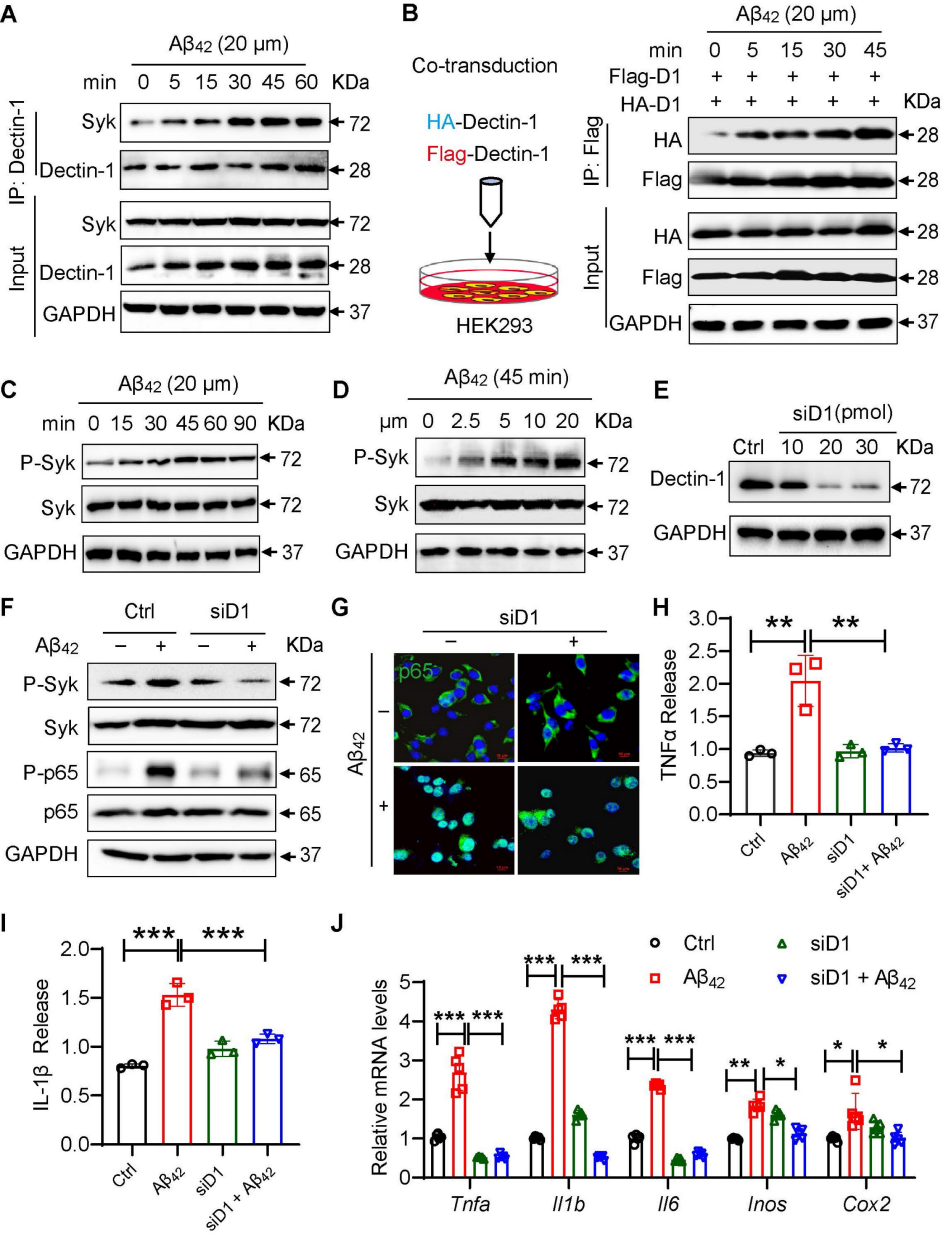
** Dectin-1 interference prevents Aβ_42_-induced inflammatory response in BV2 cells. (A)** Time-course of Dectin-1-Syk interaction. BV2 cells were exposed to 20 µM Aβ_42_ for indicated times and co-immunoprecipitation using Dectin-1 antibody to probe for Syk was performed. **(B)** HEK-293T cells were transfected with Flag-tagged Dectin-1 (Flag-D1) and HA-tagged Dectin-1 (HA-D1). Time-course of Dectin-1 dimerization (Flag-HA interaction) assessed following exposure of cell to 20 µM Aβ_42_ for indicated times. **(C)** Time-course of Syk phosphorylation. BV2 cells are exposed to 20 µM Aβ_42_ for indicated times. Total proteins were extracted and probed for p-Syk and Syk levels. GAPDH was used as loading control.** (D)** Dose-course of Syk phosphorylation. BV2 cells were exposed to increasing levels of Aβ_42_ for 45 mins. Total proteins were used to measure p-Syk and Syk levels. GAPDH was used as loading control.** (E)** Determination of Dectin-1 interference efficiency in BV2 cells. Total proteins were extracted and probed for p-Syk and Syk levels. GAPDH was used as loading control.** (F)** Representative western blot analysis of p-Syk, Syk, P-NFκB65, and NFκB65 in BV2 cells. GAPDH was used as loading control.** (G)** Representative immunofluorescence staining of NFκB65 (green) in BV2 cells transfected with or without siD1 [scale bar = 10 μm].** (H)** TNFα release in cell supernatant measured by ELISA.** (I)** IL-1β release in in cell supernatant measured by ELISA. **(J)** mRNA levels of *Tnfa, Il1b, Il6, Inos,* and *Cox2* in the hippocampus tissues. Transcript levels were normalized to *GAPDH*.

Based on the apparent functional role of Dectin-1 in AD inflammation, we explored the potential molecular mechanisms underlying Dectin-1 activation by Aβ_42_. Dectin-1 homodimerization and Syk recruitment are the two hallmarks of Dectin-1 activation. We first tested the interaction between Dectin-1 and Syk in microglia and in Aβ_42_-infused mice. The results showed that Aβ_42_ increased the interaction between Dectin-1 and Syk in a time-dependent manner in BV2 cells (Figure 5A). Increased Dectin-1-Syk interaction was also observed in the hippocampi of Aβ_42_-infused mice (Figure S7A). Next, we transfected HEK-293 cells with Flag- and HA-tagged Dectin-1 and exposed the cells to Aβ_42_. Co-IP analysis showed Aβ_42_-induced Flag-HA interaction, indicating Dectin-1 homodimerization (Figure 5B). We also exposed microglia to 20 μM Aβ_42_ for different durations to examine Syk phosphorylation and observed Aβ_42_-induced Syk phosphorylation peaking at 45 min (Figure 5C). Furthermore, Aβ_42_ challenge for 45 min induced Syk phosphorylation in a dose-dependent manner in BV2 cells (Figure 5D).

We then measured whether Dectin-1 mediated the Aβ_42_-induced inflammatory response in cultured microglial BV2 cells. We interfered with Dectin-1 expression with siRNA targeting the Dectin-1 gene (siD1) in BV2 cells and verified the knockdown efficiency by western blotting (Figure 5E). Similarly, we found that Aβ_42_ induced the Syk phosphorylation and NF-κB activation in BV2 cells, while this increased activity of Syk/NF-κB was not seen in Dectin-1-deficient BV2 cells (Figure 5E-5F). As shown in Figure 5G, knocking down Dectin-1 remarkably inhibited Aβ_42_-induced p65 nuclear translocation in BV2 cells. These results are consistent with the effect of Dectin-1 in Aβ_42_ infusion model mice. Assessment of neuroinflammation showed that Dectin-1 deficiency decreased the release of the inflammatory factors TNF-α and IL-1β (Figure 5H-5I). Moreover, the Aβ_42_-increased mRNA levels of the inflammatory genes *Tnfa, Il1b, IL-6, Cox2,* and* Inos* were significantly reversed by siD1 in BV2 cells (Figure 5J).

### Aβ_42_ directly bind to Dectin-1

**Figure 6:**
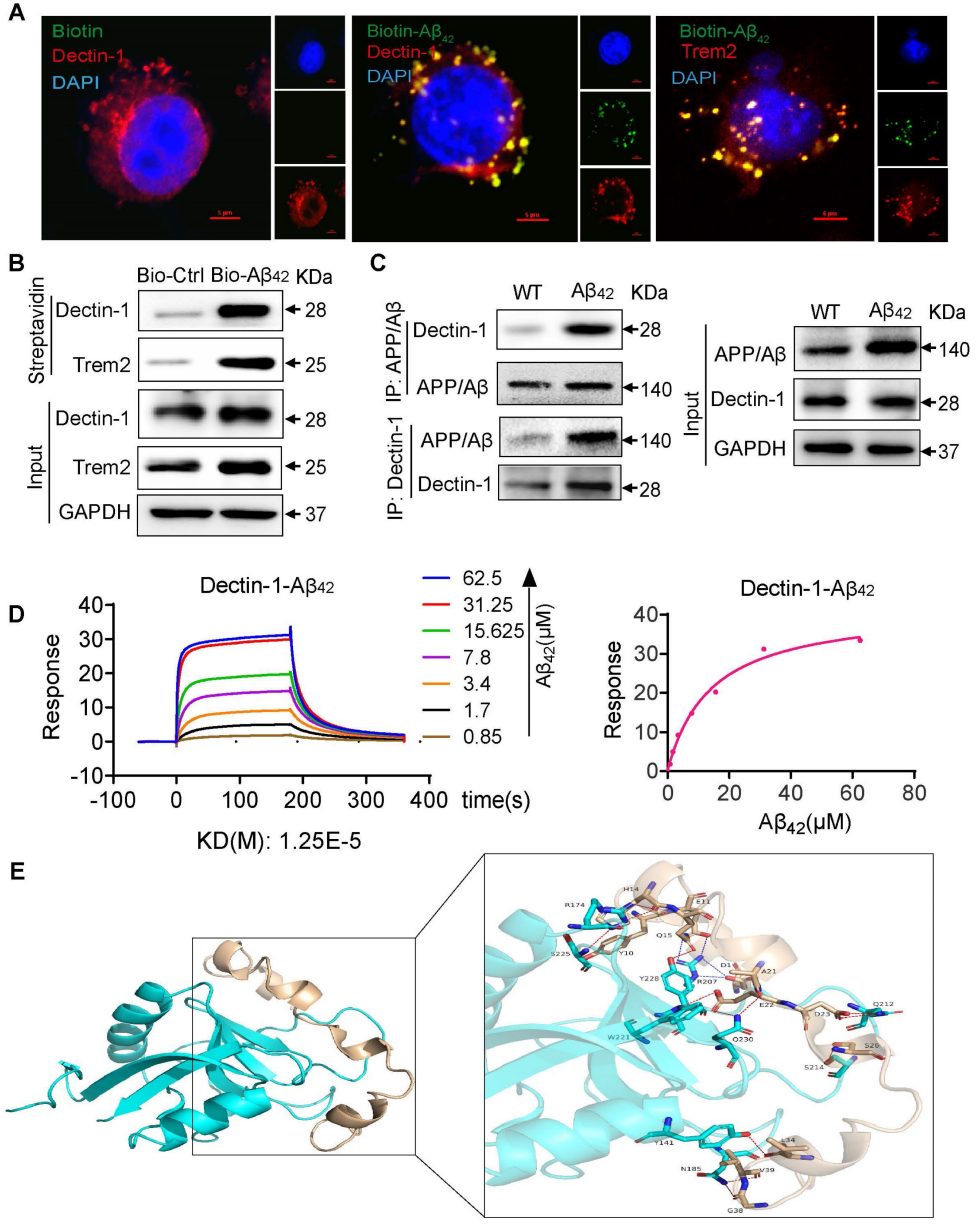
** Aβ_42_ activates the Dectin-1-Syk signaling pathway by binding to Dectin-1 and inducing its homodimerization. (A)** BV2 cells are treated with 20 µM Bio- Aβ_42_ or free biotin for 45 min, and cells are double-stained for biotin (green) and Dectin-1 (red) or Trem 2 (red), respectively. Nuclei are counterstained with DAPI (blue) [scale bar = 5 μm]. **(B)** Bio-Aβ_42_ is added to streptavidin-agarose beads and incubated. Biotin alone was used as control. Lysates prepared from BV2 cells were added to beads. The eluent was loaded onto a polyacrylamide gel for western blot analysis. Total lysates were used as input controls. Trem2 was used as a positive control. **(C)** The Dectin-1-APP/ β-amyloid interaction is analyzed by co-immunoprecipitation in the brains of WT and model mice.** (D)** Surface plasmon resonance (SPR) analysis showing a direct interaction between Aβ_42_ and rhDectin-1. Mean KD constant derived from five separate experiments. **(E)** 3D binding model of dectin-1 and amyloid beta peptide. The backbones of dectin-1 and amyloid beta peptide are shown as cartoons. The residues in dectin-1 are shown as cyan sticks, whereas the residues in amyloid beta-peptide are shown as wheat sticks. The hydrogen bonds are depicted as red dashed lines and the salt bridges are depicted as blue dashed lines.

The triggering receptor expressed on myeloid cells 2 (Trem2) is a microglial surface receptor genetically linked to the risk of AD 28. Most Aβ functions in microglia are thought to be mediated by its binding to Trem2 29. To better understand how Aβ_42_ activates Dectin-1, we visualized the molecular processes using biotinylated Aβ_42_ (Bio-Aβ_42_). Bio-Aβ_42_ retained the Dectin-1 activating activity of Aβ_42_, as evidenced by the increased phosphorylation of Syk in microglia exposed to Bio-Aβ_42_ (Figure S7B). After exposure of microglia to Bio-Aβ_42_ or biotin, we performed immunofluorescence co-staining for Bio-Aβ_42_, Dectin-1, and Trem2 and found that all of them were mainly located in the cell membrane. Trem2 is the currently reported microglia receptor that can directly bind to Aβ, and we used it as a positive control in this experiment. Interestingly, in addition to co-localization with Trem2, Aβ_42_ also co-localized with Dectin-1 significantly on the cell surface (Figure 6A), indicating a potential interaction between Aβ_42_ and Dectin-1. Co-IP analysis further confirmed this result (Figure 6B). Next, we performed a protein interaction analysis using Co-IP. APP/β-amyloid was added to Protein A magnetic beads as a bait protein, and lysates from WT and Aβ_42_ brain tissues were added. Our results showed that APP/β-amyloid binds to the Dectin-1 protein in these lysates. In turn, we could also capture the expression of APP/β-amyloid protein using Dectin-1 as a bait protein (Figure 6C). The above data prompted us to hypothesize that Aβ_42_ may act as a ligand and directly interact with Dectin-1 in the microglia. Therefore, we used surface plasmon resonance (SPR) to identify direct Aβ_42_-Dectin-1 interactions at the molecular level. As shown in Figure 6D, Aβ_42_ interacted with recombinant human Dectin-1 (rhDectin-1) protein with high affinity.

To strengthen this finding, we conducted molecular docking to analyze the interaction between Dectin-1 and amyloid beta peptide. The weighted energy score of the cluster center of complex Dectin-1 and amyloid beta peptide is -820.5 kcal/mol and the energy score of the lowest energy structure in the cluster is -908.1 kcal/mol. The interaction between Dectin-1 and amyloid beta peptide is depicted in Figure 6E. A summary of the interactions between Dectin-1 and amyloid beta peptide is presented in Table S3. The surface binding model of Dectin-1 and amyloid beta peptide is shown in Figure S8A-C. The Dectin-1 is colored in cyan, whereas amyloid beta peptide is colored in wheat. These interactions mainly contribute to the binding energy between Dectin-1 and amyloid beta peptide. Taken together, these results show that Dectin-1 can directly bind to Aβ_42._

### Dectin-1 deficiency in microglia ameliorates Aβ_42_-induced neuronal cell damage

**Figure 7:**
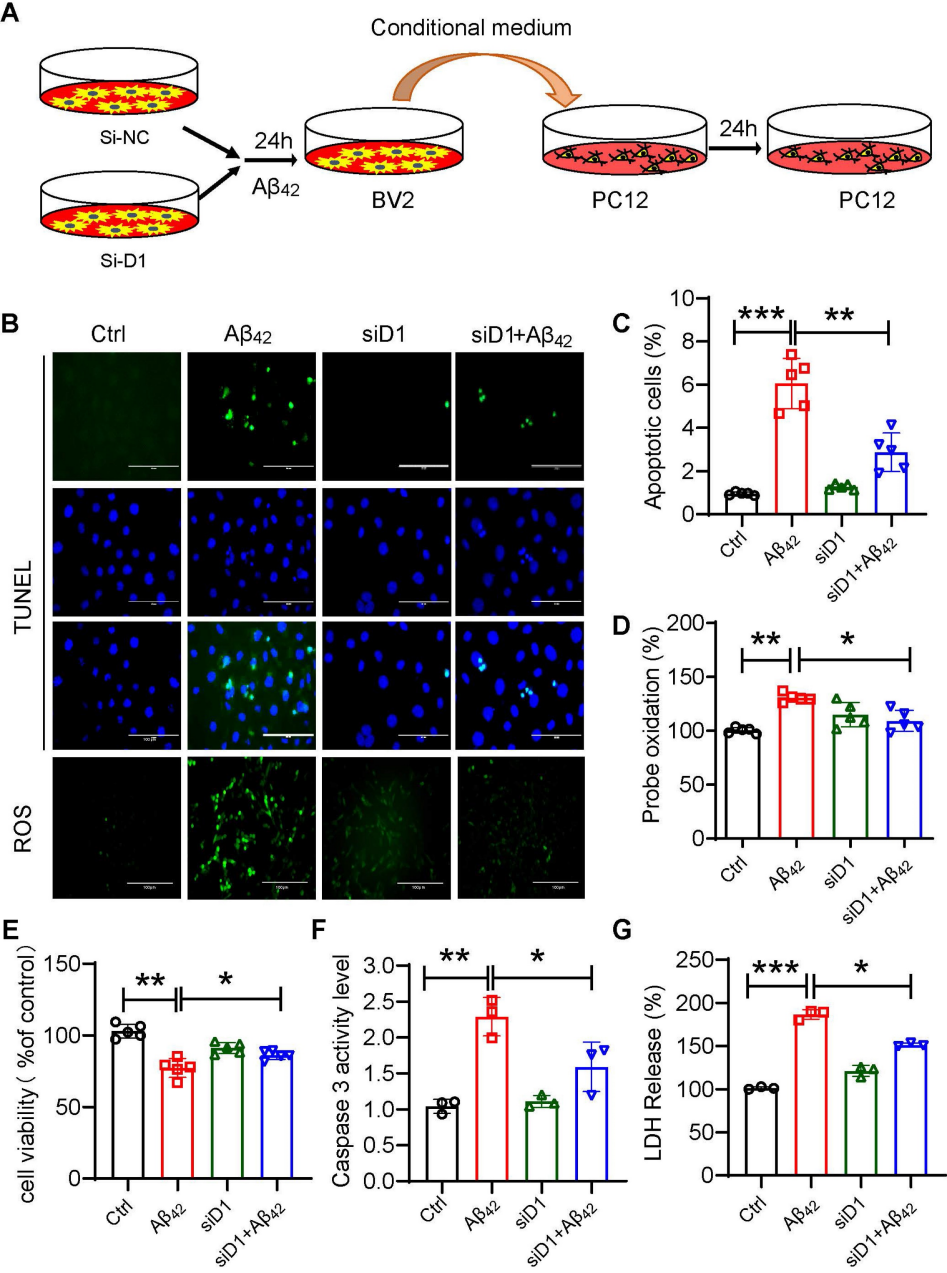
** Dectin-1 interference prevents Aβ_42_-induced neuronal damage. (A)** BV2 cells pretreated with siD1 for 24 h are stimulated with/without 20 µM Aβ_42_ for another 24 h, and the conditioned culture supernatant is collected. **(B)** PC12 cells are plated into four groups: CTL, Aβ_42_, siD1, and siD1+Aβ_42;_ BV2 serum is added to stimulate the PC12 cells for 24 h; and cell viability is measured by MTT assay. **(C)** LDH assay is used to test cell membrane damage. **(D)** TUNEL staining and Ros staining are used to test the apoptosis and intracellular ROS level [scale bar = 100 μm]. **(E)** Quantification of apoptotic cell in TUNEL staining in C. **(F)** Caspase 3 activity is detected using caspase 3 assay. **(G)** Quantification of ROS staining in D.

The activation of microglia and inflammation-mediated neurotoxicity have been suggested to play key roles in the pathogenesis of AD 30. To assess the effect of macroglia-derived inflammatory factors on neuronal cells, we prepared conditioned media from BV2 cells. BV2 cells with or without siD1 pretreatment were exposed to Aβ_42_ for 6 hours, washed with 1xPBS, and then replaced with 10% DMEM medium for 24 hours to collect conditioned medium. PC12 cells were treated with microglia-conditioned medium for 24 h to detect the damaging effect of the inflammatory environment on neuronal cells (Figure 7A). The results showed that conditioned media from microglia exposed to Aβ_42_ increased PC12 apoptosis and intracellular ROS production (Figure 7B-7G). Media produced from Dectin-1-deficient microglia failed to damage PC12 cells. Collectively, these results demonstrated that blocking Dectin-1 in microglia suppressed Aβ_42_-induced inflammatory mediators and subsequent intercellular crosstalk, resulting in reduced neuronal cell damage.

## Discussion

In this study, we discovered an important role of microglial Dectin-1 in mediating Aβ-induced neuroinflammation and AD pathology. We found that microglial Dectin-1 levels were increased in AD models. Knockout of Dectin-1 ameliorated cognitive impairment and neuronal cell death in Aβ_42_ infusion model mice by inhibiting Aβ_42_-induced microglial activation and inflammatory cytokine release. Mechanically, our studies showed that Aβ_42_ binds directly to the extracellular domain of Dectin-1 to activate the Dectin-1-Syk-NF-κB pro-inflammatory signaling pathway in microglia. Taken together, these studies have identified a new Aβ_42_ receptor in inflammatory induction and AD pathology.

Studies have shown that microglia play an important role in the development of AD 31. Microglia act as “macrophages” in the brain, which are very sensitive to brain damage 32. In the brains of AD models, β-amyloid plaques are surrounded by activated microglia 33. In-depth research on the pathogenesis of AD has shown that neuroinflammation is an important mediator of Aβ-induced neuronal death and is an important factor in AD pathology 34,35. Studies have shown that Aβ can induce a significant increase in the expression of the inflammatory cytokines TNF-α and IL-6. In the early stages of AD, the inflammatory cytokine gene-related transcription factor NF-κB can be activated by Aβ deposition 36. Several studies have revealed that Aβ stimulates pro-inflammatory activation of primary microglia 37, 38. Hong et al. have demonstrated that microglia engulf abnormal synapses when challenged with oligomeric Aβ 39. In this study, we analyzed neuroinflammation in the brain following Aβ_42_ infusion. Experimental animal models of AD can be generated by stereotaxic injection of Aβ_42_ because it induces characteristic pathological changes of AD, such as Aβ deposition, neuroinflammation, and learning and memory dysfunction 40-42. Therefore, Aβ_42_-injected animals are one of the suitable experimental models to explore the treatment of AD to study the mechanism of toxicity against Aβ_42_. As expected, we found elevated levels of pro-inflammatory factors and an increased number of microglia in Aβ_42_ infusion model mice. An imbalance in the inflammatory environment can cause neuronal cell damage. These results are consistent with those of previous studies. However, little is known about the precise molecular mechanism by which Aβ_42_ induces microglial inflammatory responses.

Dectin-1 was first identified as a PRR of β-glucans in fungal pathogens 43. Interestingly, recent studies have shown that Dectin-1 is also involved in non-pathogen-mediated sterile inflammation in addition to the classical β-glucan ligand. Dectin-1 has been reported to ligate lectin galectin-9 to promote pancreatic carcinoma immune-tolerance 14. Recently, studies have explored the biological role of Dectin-1 in brain-related diseases. Dectin-1 signaling plays a crucial role in inflammatory activation after ischemic stroke 44. In addition, Dectin-1 limits autoimmune neuroinflammation and promotes myeloid cell-astrocyte crosstalk in encephalomyelitis (EAE) 45. In the APP/PS1 mouse model of AD, Dectin-1 was upregulated by apolipoprotein E4 (APOE4) in the plaque microenvironment, suggesting that APOE4-regulated Dectin-1 expression may represent a risk protein for AD 46. These studies suggest an important role of Dectin-1 in CNS diseases. Our study is the first to investigate the role of Dectin-1 in the pathogenesis of AD. We showed that microglia Dectin-1 is overexpressed in AD model mice and that Aβ_42_ quickly activates Dectin-1 as well as its downstream signals to promote microglial activation and inflammation.

Trem2 is recognized as a cell surface receptor of Aβ in microglia 47. Studies have found that Trem2 is upregulated near Aβ plaques in AD models and is thought to respond to Aβ 48. Aβ directly binds to Trem2 and potently inhibits Aβ_42_ polymerization 49. There are many interventions targeting the Trem2 pathway as therapeutic targets for AD. However, Trem2 deficiency reduces amyloid pathology in the early stages of AD but aggravates its pathology in the late stage 50, so it is not suitable as a potential target for the treatment of AD. Dectin-1 has been reported to be highly expressed on various myeloid cells including monocyte/macrophage and neutrophil lineages 51. Our study confirmed the colocalization of Dectin-1 protein with Aβ_42_. We also found that Dectin-1 did not affect the clearance of Aβ_42_ in microglia. These data indicate that Dectin-1 is a novel and non-classic receptor of Aβ_42_ for Aβ_42_-induced inflammation in microglia. Compared to Trem2, targeting Dectin-1 may provide a new and effective strategy for the AD therapy.

Studies have identified an important role of microglia-mediated neuroinflammation in the development of AD. Our in vitro study also showed that Dectin-1 deficiency in microglia significantly reduced PC12 cell injury induced Aβ_42_-challenged microglia medium. As mentioned above, Dectin-1-mediated microglial inflammation subsequently damages the neuronal cells. We also found that Dectin-1 knockout has a comprehensive reversal effect on AD pathology, including cognitive function, synaptic plasticity, neuroinflammation, and neuronal cell injury, suggesting that Dectin-1 deficiency can be a therapeutic strategy for AD. However, some questions still need to be addressed. For example, does Dectin-1 in astrocytes and circulating macrophages also contribute to neuroinflammation and AD pathology in Aβ_42_-induced mice? Although we showed that Dectin-1 is mainly expressed in microglia in Aβ_42_-induced whole-body Dectin-1 KO mice. We acknowledge that microglia-specific Dectin-1 knockout may be more accurate. Therefore, a limitation of this study is that it was not performed using microglia-specific Dectin-1 knockout mice. In addition, another question is, besides Aβ_42_, whether other stimuli (or Aβ_42_-produced DAMPs) can activate Dectin-1 in AD and promote the development of AD. This is an important direction worthy of future research and will extend our understanding of neuroinflammation in AD. Our* in vitro* results demonstrated that Aβ_42_ could directly activate microglial Dectin-1, which supports the current conclusion of this work. Further studies are needed to illustrate the contribution of Dectin-1 to other cell types.

In conclusion, to our knowledge, this is the first study to identify Dectin-1 as a new Aβ_42_ receptor in AD pathology. We demonstrated that microglia Dectin-1 mediates Aβ_42_-induced Syk/NF-κB signaling pathway activation and inflammatory response and subsequently contributes to AD pathology and cognitive dysfunction. We provide evidence for the direct binding of Aβ_42_ to Dectin-1 and a deep understanding of the pro-inflammatory mechanism of Aβ_42_. This study suggests that Dectin-1 may represent an attractive new strategy for the treatment of AD.

## Supplementary Material

Supplementary figures and tables.

## Abbreviations

Aβ_42_: Amyloid beta peptide 1-42
Bio-Aβ_42_: biotin-labeled Amyloid beta peptide
D1KO: Dectin-1^-/-^
siD1: siRNA targeting Dectin-1 gene
IL-1β: Interleukin 1 beta
IL-6: Interleukin-6
ITAM: immunoreceptor tyrosine-based activation motif
SPR: surface plasmon resonance
Syk: spleen tyrosine kinase TAC
TNFα: Tumor necrosis factor-α
WGA: wheat germ agglutinin
Iba1: Induction of brown adipocytes
Inos: inducible NOS
Cox2: Cytochrome c oxidase subunit 2
GAPDH: glyceraldehyde 3-phosphate dehydrogenase
CLEC7A: (C-Type Lectin Domain Containing 7A
PRRs: Pattern recognition receptors
MAP2: microtubule-associated protein 2
PSD95: postsynaptic density protein 95
TUNEL: Terminal-deoxynucleoitidyl Transferase Mediated Nick End Labeling
RIPA: Radio immunoprecipitation assay
SDS-PAGE: 67 Sodium dodecyl sulfate polyacrylamide gel electrophoresis
