# Supplementary material for: β-amyloid binds to microglia Dectin-1 to induce inflammatory response in the pathogenesis of Alzheimer's disease
Source: Int J Biol Sci. 2023 Jun 19;19(10):3249–65. doi: 10.7150/ijbs.81900 (PMC10321287; doi:10.7150/ijbs.81900)
Supplement: Supplementary file 1 — Supplementary figures and tables. [file ijbsv19p3249s1.pdf]

Supplemental Figures and Tables

Table S1. Antibodies for western blot in this study

Antibody	Cat. NO	Source	Dilution
Dectin-1	ab140039	Abcam	WB:1:1000/IF:1:200
NF-κB p65	8242	CST	WB:1:1000
P-NF-κB p65	3033	CST	WB:1:1000
SYK	80460s	CST	WB:1:1000
P-SYK	2710s	CST	WB:1:1000
TNF-α	3707	CST	WB:1:1000
IL-1β（3A6）	12242s	CST	WB:1:1000
MAP2	4542	CST	WB:1:1000/IF:1:200
PSD95	2507	CST	WB:1:1000
Iba1	17198	CST	WB:1:500 /IF:1:100
Trem2	ab209814	Abcam	IF:1:200
APP/β-Amyloid	2450	CST	IF:1:200
GAPDH	AF0006	Beyotime	WB:1:2000
HA-tag	51064-2-AP	Proteintech	WB:1:500
Flag-tag	20543-1-AP	Proteintech	WB:1:500
Anti-rabbit IgG HRP	A0208	Beyotime	WB:1:2000
Anti-Mouse IgG HRP	A0216	Beyotime	WB:1:2000
Alexa Fluor® 594	8889	CST	IF: 1:500
Alexa Fluor® 488	4412	CST	IF: 1:500

Table S2. The information of reagents or resource.

**Primers**		
TNF-α	Forward	5’-GAATGCTGGTGTATAAGTCTG-3’
	Reverse	5’-TATGTCATCAACTCGGTCAA-3’
IL-1β	Forward	5’-GCAGGCAGTATCACTCATT-3’
	Reverse	5’-CAGCAGGTTATCATCATCATC-3’
IL-6	Forward	5’-CCTCTGGTCTTCTGGAGTA-3’
	Reverse	5’-ATGAATTGGATGGTCTTGGT-3’
SLC7A11	Forward	5’-ATCAGGCATCTTCATCTCC-3’
	Reverse	5’-AGACCTCCAGAATGTATGTG-3’
GAPDH	Forward	5’-TGCCCAGAACATCATCCCT-3’
	Reverse	5’-GGTCCTCAGTGTAGCCCAAG-3’
**siRNA**		
Dectin-1-Mus-316	Sense	5’-GGAGAAAUCCAGAGGAGAATT-3’
	Antisense	5’-UUCUCCUCUGGAUUUCUCCTT-3’
**Detail information of β-Amyloid (1-42)**		
Sequence (Three-Letter Code)	H - Asp - Ala - Glu - Phe - Arg - His - Asp - Ser - Gly - Tyr - Glu - Val - His - His - Gln - Lys - Leu - Val - Phe - Phe - Ala - Glu - Asp - Val - Gly - Ser - Asn - Lys - Gly - Ala - Ile - Ile - Gly - Leu - Met - Val - Gly - Gly - Val - Val - Ile - Ala - OH	
One Letter Code	DAEFRHDSGYEVHHQKLVFFAEDVGSNKGAIIGLMVGGVVIA	
Molecular Formula	$C_{203}H_{311}N_{55}O_{60}S$	
Molecular Mass	4514.10	

Table S3. The contact list between dectin-1 with amyloid beta-peptide

Chain 1	Residue	Chain 2	Residue	Interaction type
Dectin-1	Tyr141(OH)	Amyloid beta-peptide	Leu34(O)	Hydrogen bond
Dectin-1	Arg174(NH1, NH2)	Amyloid beta-peptide	His14(O)	Hydrogen bond
Dectin-1	Asn185(ND2)	Amyloid beta-peptide	Gly38(O)	Hydrogen bond
Dectin-1	Asn185(ND2)	Amyloid beta-peptide	Val39(O)	Hydrogen bond
Dectin-1	Arg207(NH2, NE)	Amyloid beta-peptide	Asp1(OD2)	Salt bridge
Dectin-1	Arg207(NH2, NH1)	Amyloid beta-peptide	Glu11(OE1, OE2)	Salt bridge
Dectin-1	Gln212(NE2)	Amyloid beta-peptide	Asp23(OD1, OD2)	Hydrogen bond
Dectin-1	Ser214(OG)	Amyloid beta-peptide	Ser26(OG)	Hydrogen bond
Dectin-1	Trp221(NE1)	Amyloid beta-peptide	Glu22(OE2)	Hydrogen bond
Dectin-1	Ser225(O)	Amyloid beta-peptide	Tyr10(OH)	Hydrogen bond
Dectin-1	Ser225(N)	Amyloid beta-peptide	His14(NE2)	Hydrogen bond
Dectin-1	Tyr228(OH)	Amyloid beta-peptide	Gln15(OE1)	Hydrogen bond
Dectin-1	Gln230(NE2)	Amyloid beta-peptide	Ala21(O)	Hydrogen bond
Dectin-1	Gln230(NE2)	Amyloid beta-peptide	Glu22(OE1)	Hydrogen bond

Table S4. Sources of Software

Software	Sources
SOAPnuke (v1.5.2)	https://github.com/BGI-flexlab/SOAPnuke
HISAT2(v2.0.4)	http://www.ccb.jhu.edu/software/hisat/index.shtml
Ericscript (v0.5.5)	http://ericscript.sourceforge.net/
rMATS (V3.2.5)	http://rnaseq-mats.sourceforge.net
Bowtie2 (v2.2.5)	http://bowtiebio.sourceforge.net/%20Bowtie2%20/index.shtml
RSEM (v1.2.12)	https://github.com/deweylab/RSEM
Pheatmap (1.0.8)	https://cran.r-project.org/web/packages/pheatmap/index.html
DESeq2(v1.4.5)	http://www.bioconductor.org/packages/release/bioc/html/DESeq2.html

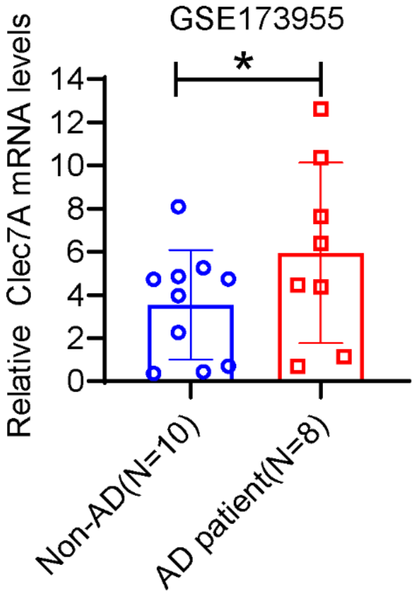

Supplementary Figure S1. Dectin-1 levels are increased in the AD patients.

The relative levels of Dectin-1(Clec7a) in Non-AD patients and AD patients, identified from a publicly available study: GSE173955. Data were analyzed to confirm findings of the present study.

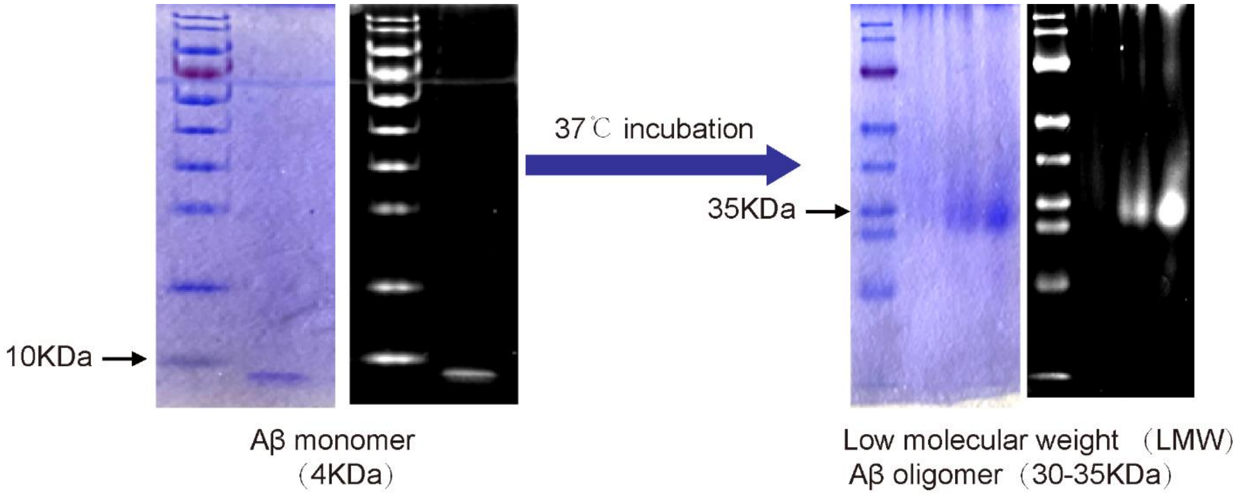

Supplementary Figure S2. Identification of Aβ oligomers.

Aβ monomer (Monomer) and oligomer (Oligomer) were detected by native-PAGE gel combined with Coomassie brilliant blue staining.

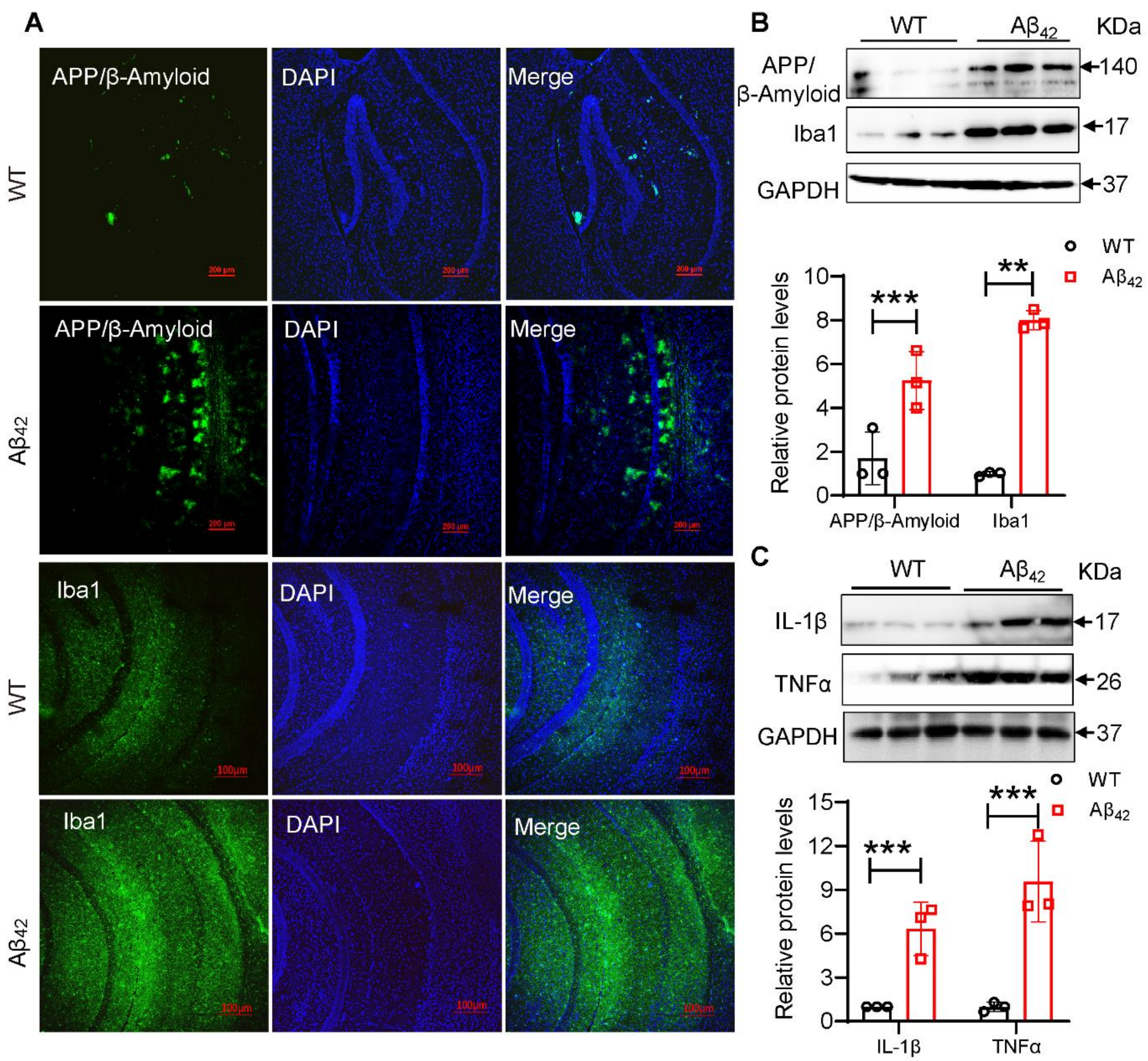

Supplementary Figure S3: Dectin-1 levels are increased in the brain after Aβ_{42} infusion. (A) Representative immunofluorescence staining of APP/β-Amyloid (red) [scale bar = 200μm], Iba1(green) [scale bar = 100μm]in brain tissues of WT and Aβ_{42} infusion mice. Sections were counterstained with DAPI (blue). (B) Representative western blot analysis of APP/β-Amyloid and Iba1 in brain tissue from WT and model mice. (C) Representative western blot analysis of TNFα and IL-1β in hippocampal tissue from WT and Aβ_{42} infusion model mice.

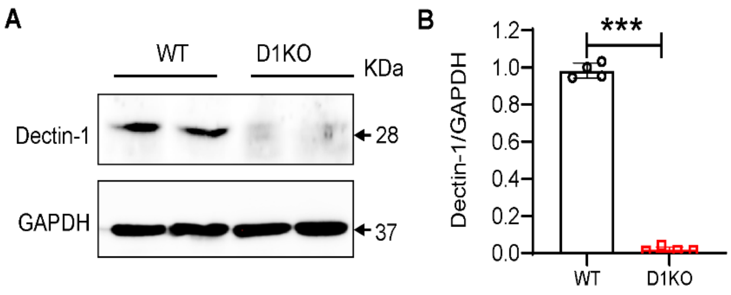

Supplementary Figure S4: Expression of Dectin-1 in the brain tissue of Dectin-1 knockout mice.

(A) Representative western blot analysis of Dectin-1 protein in brain tissue from WT and Dectin-1 knockout mice. GAPDH was used as loading control [n =4].

(B) Quantification of western blot results.

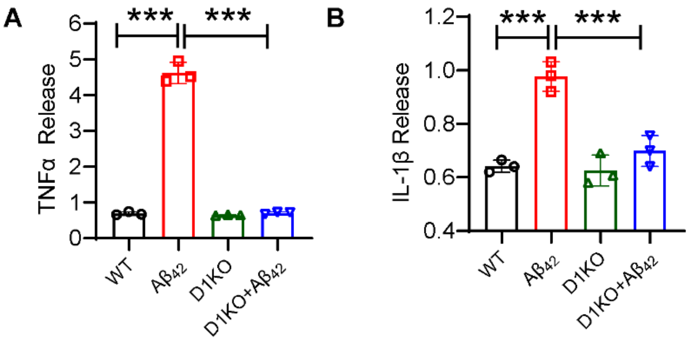

Supplementary Figure S5: Dectin-1 mediates the release of inflammatory response in brain tissue.

(A) TNFα release in brain tissues of mice were measured by ELISA.

(B) IL-1β release were measured by ELISA in brain tissues of mice.

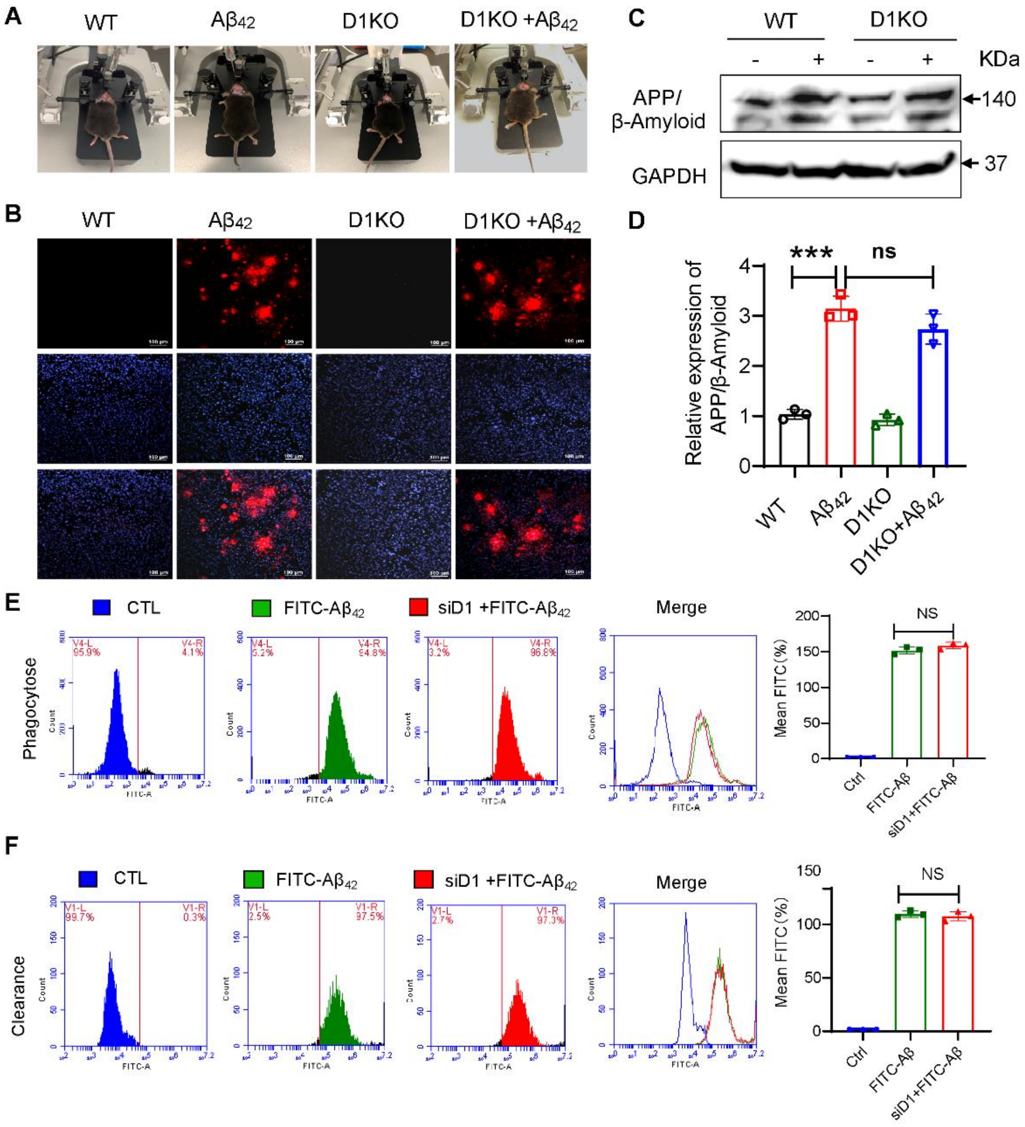

Supplementary Figure S6: Dectin-1 could regulate the phagocytic ability of microglia to Aβ42, but not affect the phagocytosis and clearance of Aβ42.

(A) Schematic of the experimental model. (B) Representative immunofluorescence staining of APP/β-Amyloid (red) in brain tissues of WT and Model (Aβ42 infusion) mice. Sections were counterstained with DAPI (blue) [scale bar = 100μm]. (C) Representative western blot analysis of APP/β-Amyloid in brain tissue from WT and model mice. (D) Quantification of APP/β-Amyloid in C. (E) Flow cytometry was used to detect the phagocytosis of Aβ42 by Dectin-1. (F) Flow cytometry was used to detect the clearance of Aβ42 by Dectin-1.

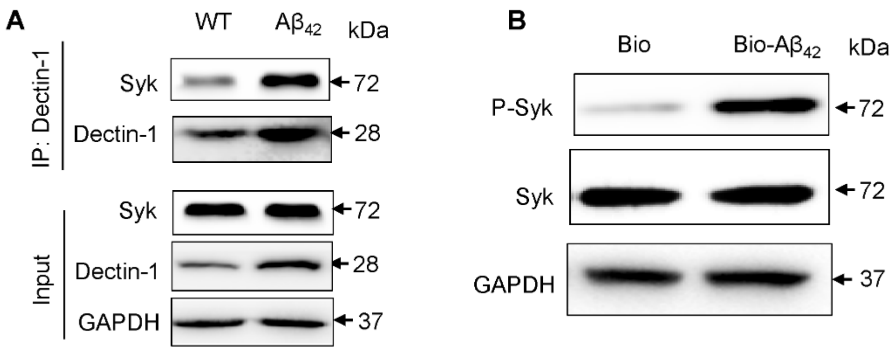

Supplementary Figure S7: Dectin-1-Syk interaction analysis in $A\beta_{42}$ model**.**
(A) Dectin-1-Syk interaction was analyzed by co-immunoprecipitation in brain tissues of WT and $A\beta_{42}$ infusion mice. (B) BV2 cells were exposed to Bio or 20 μM Bio-$A\beta_{42}$ for 45mins. Total proteins were extracted and probed for p-Syk and Syk levels were measured by western blot.

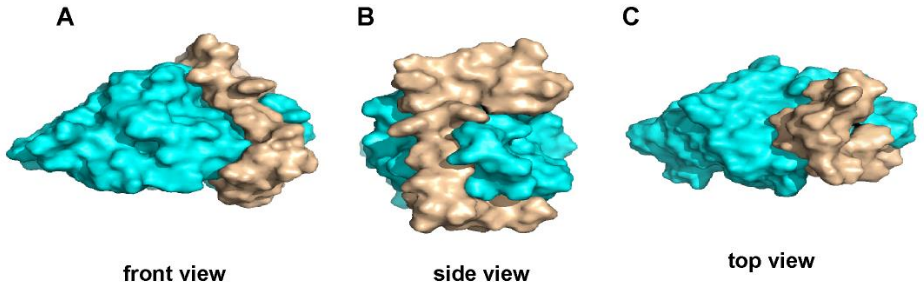

Supplementary Figure S8: The surface binding model of dectin-1 and amyloid beta-peptide.
(A) Front view of surface binding. (B) Front view of surface binding. (C)Top view of surface binding.
